# Insights into the Role of Vitamin D in the Prevention and Control of Cancer and Other Chronic Noncommunicable Diseases: Shedding Further Light on a Captivating Subject

**DOI:** 10.3390/nu16132166

**Published:** 2024-07-08

**Authors:** Alejandro Martin-Gorgojo, Jose M. Martin-Moreno

**Affiliations:** 1 Madrid City Council, STI/Dermatology Department, 28006 Madrid, Spain; alejandromartingorgojo@aedv.es; 2 Department of Preventive Medicine and Public Health, Universitat de Valencia, 46010 Valencia, Spain; 3 Biomedical Research Institute INCLIVA, Hospital Clínico Universitario de Valencia, 46010 Valencia, Spain

Vitamin D is a hormone that humans can synthesize upon sun exposure or through a balanced and healthy diet, including vitamin D-rich foods or supplements. Evidence is accumulating from animal nutrition experiments and observational and intervention studies in humans, showing that vitamin D may have valuable implications for preventing cancer and other chronic non-communicable diseases. It could influence apoptosis and tumor-associated angiogenesis, reduce the initiation of carcinogenesis, and delay the multiplication and proliferation of tumor cells. In humans, it is known that the incidence and mortality rates of some malignant tumors are lower in individuals living in southern countries, where exposure to ultraviolet light from the sun and to vitamin D itself is relatively higher. Moreover, several observational and interventional studies have been conducted, suggesting that vitamin D can help reduce the risk of cancer in people. Nevertheless, the results have been inconsistent so far. Overall, the evidence is not solid or comprehensive enough to establish that vitamin D supplementation can prevent cancer. At the same time, in a complementary manner, the possibility of associated beneficial effects has also not been ruled out. Furthermore, increasing evidence indicates that vitamin D is also crucial in both preventing and managing various chronic non-communicable ailments (NCDs), including cardiovascular diseases, diabetes, and osteoporosis. 

Driven by the unresolved issues outlined previously, this Special Issue of *Nutrients* aims to bring together research to define the state of the art and knowledge, shedding further light on the prevention and control of cancer and other NCDs. Entitled “Insights into the Role of Vitamin D in the Prevention and Control of Cancer and Other Chronic Noncommunicable Diseases”, the Special Issue includes six articles from different research groups that we, as Guest Editors, briefly summarize below.

The paper by Dr. Fukuzato et al. [1] investigates the interaction between vitamin D supplementation and marine *n*-3 polyunsaturated fatty acids (PUFAs), such as eicosapentaenoic acid (EPA) and docosahexaenoic acid (DHA), on the prognosis of digestive tract cancer patients. Examining data from a randomized controlled trial of 302 patients, the study focuses on the effects of serum levels of EPA and DHA, categorized into higher and lower halves based on median levels. The findings demonstrate a significant association between higher serum levels of EPA and DHA and improved 5-year relapse-free survival (RFS) rates. Notably, in patients with lower EPA and DHA levels, vitamin D supplementation significantly increased the 5-year RFS compared to the placebo group, highlighting a potential interactive effect between vitamin D and marine *n*-3 PUFAs. This interaction suggests that vitamin D supplementation might particularly benefit patients with lower levels of marine *n*-3 PUFAs, offering a promising approach for enhancing the prognosis of individuals with digestive tract cancers. 

The manuscript by Dr. Ismail and colleagues [2] performs a systematic review and meta-analysis to ascertain the global prevalence of vitamin D deficiency and insufficiency among patients with Multiple Myeloma (MM). The study synthesizes data from eighteen studies, revealing that the occurrence of vitamin D deficiency and insufficiency in MM patients stands at 39.4% and 34.1%, respectively. Interestingly, the analysis suggests that newly diagnosed MM patients exhibit higher rates of vitamin D deficiency and insufficiency compared to those undergoing treatment. The systematic review includes data pooled from studies conducted across several continents, including North America, Europe, Asia, Australia, and Africa, indicating a widespread prevalence of vitamin D inadequacy among MM patients globally. The authors advocate for the incorporation of vitamin D testing as a critical component in the clinical evaluation of MM, given the substantial correlation between vitamin D levels and MM patient outcomes. 

Dr. Lejman-Larysz and her team [3] investigate the influence of vitamin D on the incidence of metabolic syndrome and hormonal balance in patients with polycystic ovary syndrome (PCOS). This study, conducted on 120 women aged between 18 and 42 years, aims to address the unclear relationship between serum vitamin D deficiency in PCOS patients and the effectiveness of vitamin D supplementation. Participants were divided into two groups: those diagnosed with PCOS and a control group of regularly menstruating women without PCOS. Through detailed examinations and laboratory tests assessing various hormonal levels and vitamin D concentrations, the study found that a significant portion of PCOS patients exhibited deficient or suboptimal serum vitamin D levels. The research highlighted a correlation between vitamin D levels and several metabolic and hormonal parameters, including body mass index (BMI), waist-to-hip ratio, waist circumference, blood pressure, sex-hormone-binding globulin levels, and free-androgen indices. 

The manuscript by Dr. Ruiz-García and colleagues [4] presents a systematic review and meta-analysis of 80 randomized clinical trials (RCTs) assessing the impact of vitamin D supplementation on mortality and cardiovascular outcomes in adults. This comprehensive analysis, spanning studies published between 1983 and 2022, includes a total of 163,131 participants, with a mean age of 66.1 years and 68.6% female participants. The findings reveal that vitamin D supplementation is associated with a lower risk of all-cause mortality, with an odds ratio (OR) of 0.95, indicating a statistically significant decrease in the risk of death from any cause. However, this supplementation does not show a statistically significant association with a lower risk of cardiovascular mortality, non-cardiovascular mortality, myocardial infarction, stroke, heart failure, major adverse cardiovascular events (MACEs), or extended MACE outcomes. The study underscores the complexity of the relationship between vitamin D supplementation and health outcomes, highlighting the need for further research to explore potential benefits in specific populations and under various conditions. The evidence supports vitamin D supplementation for reducing all-cause mortality risk, particularly in fair- and good-quality RCTs, but does not suggest significant cardiovascular benefits. 

The article by Dr. Törzsök et al. [5] investigates the impact of isochromosome 12p (iChr12p) formation on vitamin D metabolism in testicular cancer. Recognizing iChr12p as a hallmark of nearly all invasive testicular cancers, the study explores how this genetic alteration might influence the disease process through dysregulation of the vitamin D metabolic pathway, which is crucial for cellular homeostasis. The research utilizes RNA sequencing analysis of vitamin D receptor (VDR) genes and examines mRNA expression of vitamin D regulatory genes in testicular germ cell tumors (TGCTs), distinguishing between pure seminomas and non-seminomatous germ cell tumors. The findings indicate that iChr12p formation could disrupt vitamin D metabolism, contributing to testicular carcinogenesis by increasing fibroblast growth factor 23 and parathyroid hormone-like hormone expression. These alterations could lead to hypercalcemia and inactivation of the VDR, potentially fostering an environment conducive to cancer development. The study suggests a complex interplay between genetic alterations in TGCT and disturbances in vitamin D and calcium homeostasis. The authors propose that iChr12p formation and vitamin D metabolism disturbances play a role in the pathogenesis of testicular cancer. Their findings support the potential for these mechanisms to be targets for future therapeutic strategies or diagnostic tools, underscoring the importance of vitamin D in testicular health and disease. 

Finally, the paper by Dr. Yang and colleagues [6] examines the association between vitamin D supplementation during Intensive Care Unit (ICU) stays and the outcomes of critically ill patients with sepsis. Utilizing data from the Medical Information Mart for Intensive Care IV (MIMIC-IV) database, the study encompasses 3539 patients with sepsis, of whom 315 received vitamin D supplementation during their ICU stay. The primary outcomes focused on all-cause in-hospital mortality, alongside 28-day and 90-day mortality rates after ICU admission. Employing propensity score matching, inverse probability of treatment weighting, and overlap weighting analyses to minimize selection bias, the study found that vitamin D supplementation was associated with significantly lower in-hospital, 28-day, and 90-day mortality rates in sepsis patients. Furthermore, multivariate regression analysis underscored vitamin D supplementation as a potentially protective factor against mortality, though no significant impact was observed on ICU or hospital length of stay (LOS). This comprehensive investigation suggests a beneficial effect of vitamin D supplementation on the prognosis of sepsis patients during ICU stays. 

The six contributions presented herein are not only highly stimulating but also offer readers compelling and pragmatic insights. Furthermore, it is evident that further exploration of this captivating topic is imperative to address lingering uncertainties and develop more efficacious practical solutions and applications. Pursuing this avenue of inquiry is not only worthy but also indispensable for the advancement of the field. 
